# The new institutional economic approach to land development game: Integrating transaction cost and efficient institutional framework in synergic urban land use-transport development model

**DOI:** 10.1016/j.heliyon.2022.e12793

**Published:** 2023-01-02

**Authors:** Anutosh Das, Sumita Roy

**Affiliations:** a Department of Urban & Regional Planning, Rajshahi University of Engineering & Technology (RUET), Rajshahi, 6204, Bangladesh; b Department of Urban Planning and Design, The University of Hong Kong (HKU), Hong Kong

**Keywords:** Transit-oriented development (TOD), Transaction cost, Property development process, Institutional arrangement, Land value capture

## Abstract

Land and property development process includes a series of multifaceted activities ranging from purchasing to converting it for development purposes and everything in between. The process itself encompasses multiple stakeholders, drivers, and contributions from diversified public and private actors and transaction cost arises out of their complex interaction. Transaction costs incurred during any kind of human interaction (i.e., transactions). Every actor involved in the process wishes to maximize his achievement under various constraints and hence institutional arrangement (i.e., set of humanly devised rules to administer the constraints) is necessary for efficient management of the development process. Therefore, to devise an optimum outcome out of economic and social transactions in the property development process, cooperative and competitive relationships between individuals should be understood from a broader socio-political and governance structure. In this research, it is critically argued that the land and property development process should implicate a multifaceted set of formal and informal rules or institutional arrangements to govern the intrinsic interaction, action and thereby reducing the related transaction cost. The central argument is further reproachfully evaluated and implicated in the urban development process through the myopic lens of the Transit-oriented development (TOD) pathway. A vigilant combination of descriptive and explanatory research approaches is adopted to analyze the connection between theory and practice.

## 1 Introduction

Land always plays a very perilous role in the socio-economic, politico-cultural, and environmental sectors for nurturing the development of people and states. For ensuring the convenience and welfare of urban dwellers, land use planning has widely recognized the integration of land use-transportation synergic relationship as a precondition for inclusive and efficient transformation of the urban built environment. In practice, it may take different forms to offer relief from the array of torment concerns arising out of the growing ribbon pattern of development and overarching motorized private transport dependency in many western countries. As a comprehensive measure in land-use planning, Transit-Oriented Development (TOD) promotes a “people-centric” approach to stimulating inclusive and integrated development for all through improved mobility and access. TOD is a mixed-use, compact and desirable built form in contemporary cities for land use planning integrating the comprehensive mass transit ridership. It eventually stimulates tremendous social and economic benefits contributing to the sustainable growth of a city and thereby the idea has attracted increasing popularity in many western cities. The improved transport accessibility consequently upsurges the land value and the captured value can be utilized to finance the urban mass transport infrastructure system and sustain its operational viability [[1], [2], [3], [4]].

But obtaining the synergy effect efficiently in an urban context is not an easy task. The land development process most often results in power and identity-related crisis followed by consequent conflicts arising out of the involvement and interaction among a wide range of diversified actors and stakeholders [5,6]. It necessitates numerous interdepend decisions, actions and interactions between different stakeholders, exchange of information, and assets of economic values, which may not be easily divisible. The concept of Transaction cost furthermore contributes to the theory of property development as a complex process. Every player involved in the process of property development wishes to achieve some objectives determined individually or collectively. Additionally, non-compliance and opportunism arising out of manmade arrangements among different players eventually tend to engender transaction costs (i.e., negotiation, and enforcement costs under divisible constraints of the process). As higher transaction costs are positively correlated with the inefficiency of the system, an efficient development process requires a considerable reduction in the cost of the transaction [4,[7], [8], [9]].

Hence, land and property development should be addressed and governed through a comprehensive human interaction management approach with due focus on the associated constraints in a local socio-economic and political context. Formulating, interpreting, and enforcing formal and informal rules or efficient institutional arrangement is indispensable in finding an efficient governance structure as well as dominant rules to govern the action and interaction of different players involved in the land development process. The effective rule structure or institutional framework correspondingly assists in identifying an effective coordinated action among numerous players and curtailing the associated transaction costs toward accomplishing a desirable and efficient outcome in transforming the urban built environment [4,10,11].

Existing literature pays limited attention to understanding the efficient institutional framework under the new institutional Economics (NIE) approach integrating Transaction cost analysis. Several researchers have tried to investigate the origins and effects of changes in the institutional setting of diversified TOD practice case studies. However, careful attention needs to fill this knowledge gap focusing on formulating, interpreting, and enforcing the Institutions (rules of the land development game) in governing the integrated and complex Transit-oriented urban land use-transport development process.

Therefore, this particular study has tried to empirically respond to the two key research questions and guide the analysis: (1) How do the formulation, interpretation and enforcement of the Rules or institutional arrangement can govern the interaction and action of various players in the land development process? (2) What are the dominant rules for efficient management of the development process concerning the Transit-oriented development (TOD) pathway in practice? In essence, this paper applies the extensive theoretical insights of new institutional economics and transaction cost framework in investigating the research argument that whether the efficient rule structure or institutional arrangement can bring anticipated development outcomes through governing the interaction and action of various players involved in the process of land and property development.

## 2 Theoretical understanding of the concept

### 2.1 Transit-oriented development (TOD)

Today, due to growing concern for broader sustainability and environmental issues, urban planners, and policymakers are more apprehensive about the drawbacks of auto-oriented cities. Several studies have acknowledged that high-density mixed-use development would generate a larger number of mass rapid transit ridership [1,12]. Cervero and Kockelman [13] have identified three critical land-use components i.e., Compact high-density built environment, diversity of land uses and pedestrian-responsive urban design that can encourage non-motorized travel reducing vehicular trips. Therefore, to address the negative externalities arising out of rapid urbanization, a renaissance transit-oriented development (i.e., mixed-use, higher-density urban development along major transport routes and nodes) has been ascertained worldwide as one of the most efficient means of urban form in contemporary cities [3].

Given the highly expensive transit infrastructure to build, maintain and operate, it is quite difficult to promote and prioritize TOD in cities with a low resource base. To overcome this challenge and associated financial obstacle, a wide array of literature has supported the use of a development-based land value capture (LVC) mechanism to capture the increased property values arising out of improvement in the transit infrastructures. The generated revenue can be utilized in turn to finance and maintain the transit infrastructure as well as provide different support services to low-income and needy groups. On general occasions, Government can sell or lease the appreciated land or development rights due to transit investment to the land developers, private authorities and transit agencies as well as can mediate partnerships among them for the transaction of property, financing, building, and maintaining the transportation infrastructures and developed properties. In this process of creating and sharing values, the developers or transit agencies can invest in transportation infrastructure development (i.e., transportation links and fixed facilities as Stations) which will eventually attract more people to do business and live there due to increased accessibility with a consequential land value increase to recover the cost of the investor as well as making handsome profits [2]. In a more complex setup like land consolidation, readjustment and urban redevelopment, the individual landowner can make small groups and consolidate their land to join a partnership with private developers in multi-purpose projects as a shareholder to make an extra profit rather than only selling land to them [3].

### 2.2 R + P model

**Figure 1:**
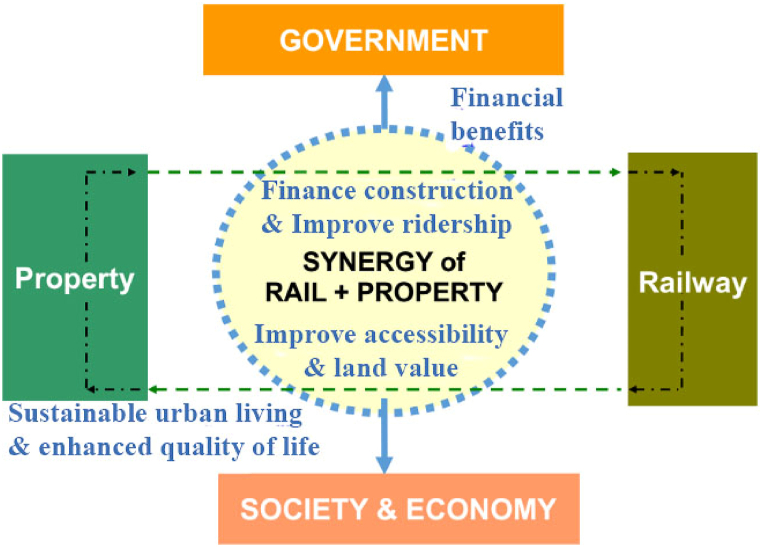
R + P model: a win-win situation for transport-land use synergy. (Source: Adapted from [[1], [2]]).

Apart from land value capture, Tang et al. [1] have identified multifaceted benefits (i.e., improved accessibility, compactness of urban development, diversified socio-economic benefit) arising out of the transport-land use synergy integrating urban mass transit railway and high-density property development near the station areas. The Synergic relationship of the integrated Railway and Property Development Model (R + P model) is exhibited in Figure 1. The R + P model is a realistic variation of TOD. It is both an “approach” and a “product”. Structures atop subway stations are the tangible outcomes of R + P. R + P is more than an end‐product of “brick and mortar” atop subway stations. It's also a well-thought-out procedure for planning, monitoring, executing, and managing station area development, as well as profiting from the resulting land price rise [14]. Tang et al. [15] have identified four key elements behind the R + P approach and those are policy, process, project, and organization. The R + P model is one of the greatest examples of how to finance railway investments using the “value capture” approach. Intensification of property around the transport nodes can support more residents through enhanced floor space as well as the sophisticated intensity of urban activities with improved accessibility. It will, in turn, improve the mass transit ridership, and more economic return and hence releases the government's burden for subsidizing the operational viability and improved efficiency of the mass rapid transit (MRT) (i.e., Railway) and human activities. When the “3D” model (density, diversity, and design) is successfully performed, transit value capture (e.g., R + P) advances both financial and wider urban growth objectives. This is a win-win situation with successful public-private cooperation: the railway corporation benefits financially, while society benefits from more sustainable, transit-oriented development patterns [14].

### 2.3 New institutional economics

Depending upon specific circumstances, different institutional forms or models such as “cooperative exchanges” or “exploitative appropriation” can govern the efficient production of the urban built environment [10,11,16]. An institutional setup like co-operative exchanges focuses on the enhanced economic efficiency or benefits of the stakeholders while welfare reduces other contracting parties through exploitative appropriation. The collective outcome of these strategic interactions, in the long run, can strengthen or transform the prevailing institutional setup governing the land development process [4,17,18].

The underlying institutions in connection with the property rights system determine whether a sensible use and development of the land resources can generate and capture the expected benefit. The new institutional economic (NIE) perspective recognizes “institutions” as the “rules of the game” for the land development process covering formal rules, informal norms, and their enforcement characteristics [7,8]. The institutional setup structures the pattern of social interaction providing the systems of incentives and constraints which impact the behavioural aspect. Moreover, this NIE approach referring to the use of neoclassical economic theories in explaining economic and social institutions has identified that market transactions and their enforcement arising out of privatization of valuable land resources and its voluntary trading with others will impose “transaction cost” [4,19].

**Figure 2:**
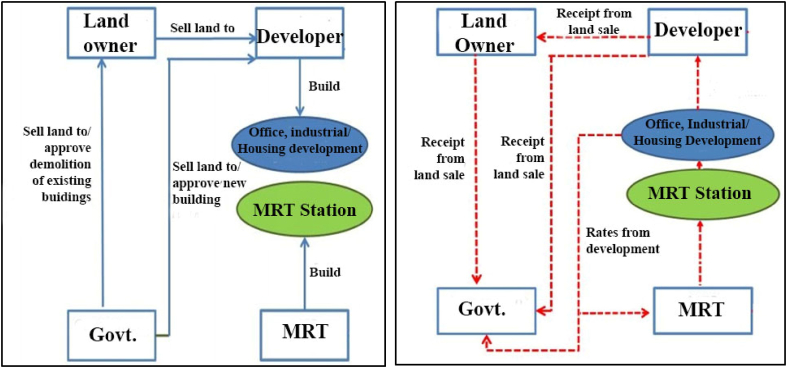
Key actions of different players (left) and the resulting cash flow (right) in the land development game. (Source: adapted from [20]).

There are several key players involved in the land Game (e.g., Landowner, Developer, government, private Transit Company and so on). Each player has some specific objective that he wants to optimize. The landowners seek to make money from the land transaction. The land Developers' sole objective is to make a handsome profit from the development process. Governments are both the planning and land authorities with multiple objectives. As the planning authority, the government is responsible for preparing the statutory plans, policy objectives and regulatory measures (i.e., formulating statutory plans, approving demolition and development, selling Government lands) to govern the actions of the players involved in the development process. Transaction costs are exerted out of the player's interaction and actions under certain constrained and opportunistic contextual situations [20]. The key actions of different players and the resulting cash flow out of a monetary transaction in the process are exhibited through an empirical illustration as shown in Figure 2.

### 2.4 Transaction cost

In general, “Transaction costs” refer to all costs which emerge due to assumed imperfect rationality and incomplete information in many neo-classical models [9,21,22]. The concept of Transaction cost was first devised in Coase's work (1960) fundamentally departing from neoclassical economics with a focus on the costs of market transactions and the efficient allocation of property rights [23]. In the new institutional economics, a transaction is assumed as the basic unit of its analysis. Dixit [24] defined it as the interchange of resources, assets of economic values, or reciprocal promises and actions between the contracting parties in society. It does not contribute directly to the output of a development process i.e., production costs. From a cost-efficiency perspective, it can be seen as deadweight losses or frictions affecting effective resource allocation and hence be minimized for a more smooth and more efficient development process through devising an appropriate institutional arrangement towards plummeting the transaction cost itself [4,25].

## 3 Review of relevant literature

Despite being comparatively a new concept, the US successfully applied the “integrated rail-property development model (R + P model)” and value capture over a century ago to finance urban streetcar networks. Currently, in US Washington Metrorail's joint development program collectively produce a very insignificant percentage of the system's annual revenues through air-rights leases and station connection fees. Further major international applications of are R + P model includes but are not limited to Rail-Integrated Projects in London (i.e., King's Cross Station, Euston Station), Redevelopment of Hudson Yards in New York, High-Speed Rail from Sydney to Melbourne, metro in Hyderabad, India and so on. These public and private funded urban rail projects highlight that innovative financial mechanisms, notably land-based mechanisms, are required to sustain and revive the urban rail integrated projects and gain financial benefits with other co-benefits [26].

In Tokyo and other large Japanese cities, there is a long tradition of private companies bundling together railway and new town development. Private railway and real-estate businesses are now cooperating with government organizations to reconstruct urban areas around key central city stations as property development has become more competitive and riskier. Building R + P buildings using transit-oriented design concepts have societal advantages that include not just significant financial returns but also thriving real estate markets and a ridership bonus [14].

Singapore has rapidly built its world-class railway network, partly supported by high automobile-related charges and land-related taxes, fees, and sales, and partly through transfer payments from the consolidated national account. Density incentives have been used in Singapore to encourage dense construction around stations and properties within MTR's catchment have enjoyed 5–15% of plot‐ratio bonuses [14]. The locations of Singapore's new town centers' rail transit stations have gradually developed into a particular TOD land use concept for Asian transit villages [27]. In Malaysia, to promote the idea of sustainable development in city development, Transit Oriented Development (TOD) integrates land use and public transportation facilities [28].

**Figure 3:**
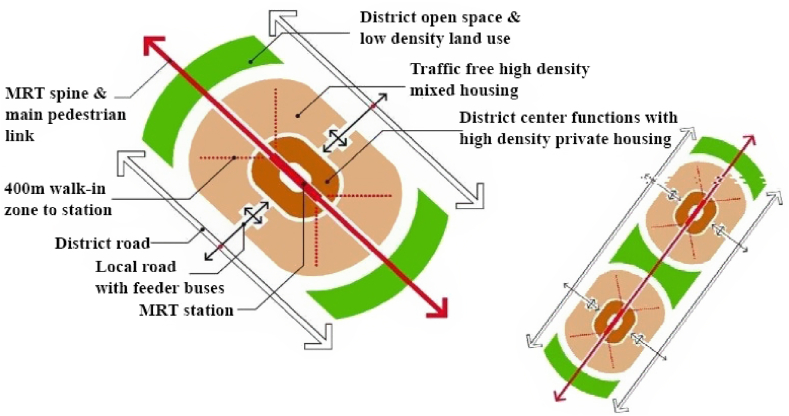
R + P supporting transit-oriented development (TOD) in Hong Kong. (Source: [2]).

Hong Kong still practices the model as an efficient means in the public transport industry as a functional instrument in guiding urban development as demonstrated in Figure 3 and hence established a unique success story with blazing international recognition of the R + P model's application throughout the world [1,12,14,29].

Rather than simply juxta-positioning railway and property development as well as using the generated real estate income for financing the development process, the R + P model embodies a unique approach i.e., institutional framework for handling and coordinating numerous players actively involved in the complex transit-oriented urban development process to achieve an efficient outcome out of land use-transport interaction [4,30]. It serves the broader purpose of town-planning objectives e.g., promoting transit-oriented development (TOD) alongside acting as a financial model [31].

## 4 Research methodology

This research has forwarded with the following null and Alternate Hypotheses. A systematic review of the concerned grey literature and meta-data-analysis approach has been primarily adopted for this research to test the hypothesis and probe the central argument of this research.

Null Hypothesis (H_0_): Land and property development process should not implicate a multifaceted set of formal and informal rules or institutional arrangements to govern the intrinsic interaction, action and thereby reducing the related transaction cost.

Alternate Hypothesis (H_1_): Land and property development process should implicate a multifaceted set of formal and informal rules or institutional arrangements to govern the intrinsic interaction, action and thereby reducing the related transaction cost.

A systematic review gathers all relevant research on a specific topic and design, and then evaluates and synthesizes their findings. A systematic review aims to compile all available empirical research using well-defined, systematic procedures to address a specific issue [32]. Systematic reviews are well-organized and follow a set of guidelines. A systematic review is a review that includes i) research question, ii) sources that were searched, iii) inclusion and exclusion criteria, iv) selection (screening) methods, and v) critically appraises and reports on the quality/risk of bias of the included studies and vi) information about data analysis and synthesis that allows the reproducibility of the results [33]. This research followed the guidance of the systematic review method for analyzing the hypotheses and coming to a conclusion.

Again, the study of “processed data” from chosen qualitative research studies to generate a systematically formed, integrated body of knowledge about a certain phenomenon is known as meta-data analysis [34].

After selecting the research problem and thus the research hypotheses following the systematic review strategy, the research forwarded with an approach to analyzing the connection between theory and practice. On this central argument, different grey literature on the theoretical concepts has been searched and after screening with the inclusion criteria some literature has been identified that deals with the key points of this research. With the meta-data-analysis approach, the literature has been analyzed on how they built the connection between theory and application of the theory and what outcome came from that connection. In this process of analyzing the central argument with the extensive theoretical insights of new institutional economics and transaction cost framework in investigating the research, the argument is reproachfully evaluated and implicated in the urban development process through the myopic lens of the Transit-oriented development (TOD) pathway.

## 5 Institutional arrangement in the land development process: empirical findings and discussions

The Land Development process as integrated railway and property development involves numerous exchanges of information and actions between many players engendering numerous ex-ante (i.e., costs involved in searching for the best deal, negotiation costs and so on) and ex-post (i.e., costs incurred after securing a contract as costs of enforcement, monitoring etc.) transaction costs. In a general setup, complying with planning regulations (e.g., hiring consultants, and extensive negotiating with the government), opaque legislative requirements, strategic choices of transacting parties, uncertainty and opportunism in the real world, bounded rationality incurs transaction costs of the development process. Efficiency rules involve a combination of institutional arrangement as assigning rights to the most capable decision agents taking complete responsibility for benefits and costs subsidiarity, the opposite orientation of incentives and constraints and internalization of effective governance of contractual relations [23,35,36].

The transaction costs framework can govern the comparison among different institutional arrangements as an effective device for coordinating the development process (9). The issue of property rights is obvious when the concept of new institutional economics and transaction cost are applied to the land development process. The success of efficient market transaction of property right highly depends on the institutional arrangement of the land and property development process. “Coase Theorem” and “New institutional economics” have suggested that privatization, market negotiations and transaction between individual parties in an open market and market transactions for these scarce land resources will lead to an optimum solution curtailing the associated conflicts and the high transaction cost as well [10,11,22,37,38].

To govern the actions and behaviour of the parties involved in the transaction, different models for the institutional arrangement or a combination of them may prove successful. Tang et al. [1] have proposed two distinguished models of the institutional form of land use to serve these purposes. The first model has focused on the statutory framework to regulate third parties’ actions (i.e., diminution, and restrictions of private individual property rights in and near transit stations) and governance of public-sector decision-making in broader planning issues. Given this model, widespread government regulation and policy measures, statutory provision of town plans, land use restrictions like zoning, and land leases are proposed to be widely practiced for ensuring coordination among key players involved in the land development process. The transit company under private ownership will act partially as a developer of the transit links and fixed facilities only. The nature of interaction among key market players as well as their compliance with the rule structure apportioned by the government policy regulations will largely determine the accomplishment of project implementation.

On the contrary, unlike the over-emphasis on the government policy restriction in the first model, the central thesis of the second model is to put special prominence on the existence and key performance of a liberal private transit company at the centre of planning and organizing the overall development process. The transit company with exclusive development rights of the transit station is proposed to govern all negotiation and conflict resolution among inter-governmental departments, land developers and other key actors to internalize all possible external benefits of the TOD projects. The central thesis of the new institutional economics is to ascertain the appropriate institutional form of governance for the transformation of the urban built environment through minimizing transaction costs from an array of institutional arrangement options like adopting public sector emphasis as demonstrated in model A or through integrated private sector performance in land use planning as proposed in model B. Therefore, International best practices and overarching case studies approach can be administered to find out empirical evidence of an efficient institutional framework for successful rail-transit integration and urban development. Furthermore, it will provide support for the mutilated theoretical explanation of property development as well as examine the dominant role of interaction among different players involved in the process [1,5,39].

## 6 Practical explanations: unique case of Hong Kong

Hong Kong demonstrates an exceptional example of a highly profitable public transport system without government subsidies. Hong Kong showcases a wide variety of R + P projects primarily focusing on housing and commercial development. In a high-density complex urban setup, several agglomeration benefits have resulted due to the successful implementation of the R + P model by the MTR Corporation (MTRC). It is a privately owned city's largest company that operates world wide's most successful build–operate–maintain (BOM) transport systems applying the ‘transit value capture’ principle of the R + P model to finance railway investments. Under this institutional setup, a public-private partnership is apprehended where the government sets major policies and creates a favorable incentive and restriction regarding the joint development of transit nodes and nearby properties. The other core market players (i.e., land developers) take an active part in project implementation as per the set rules of the joint agreement while pursuing their self-interest. The MTRC acts as a mediator between the key market players and the government for coordinating the joint venture project implementation and balancing possible conflicts between public and private interests. It also helps to reduce the complex transaction costs that emerged over the long project duration by diminishing the intermediaries within the institutional setup in the whole development process [1,31].

As an organized institutional setup, for building and operating the railway MTRC does not receive any direct cash subsidy from the government of HKSAR (Hong Kong Special Administrative Region) but rather receives a land grant in form of exclusive development rights for the land on and adjacent to the station. Both parties benefited from the transactions. The government has not to pay extra subsidies for transport infrastructure development and receives extra benefits for additional floor space generated through R + P models to contribute toward the growing housing demand in Hong Kong. Private residential developments have been drawn to the area since accessibility has improved as a result of the MTR network expansion [40]. The MTRC uses the value captured through transit development utilizing the real estate development potentials of its stations. The MTRC has been effective in controlling operating costs, including staff costs, energy costs, repairs and maintenance, and other expenses, with earnings from property development representing one of the most significant portions of the overall return on investment [41]. Moreover, receiving the land grant from the HKSAR government relieves MTRC from buying precious land in Hong Kong from the private property market at a sky-mounting price. At present, MTRC generates the lion's share of its income from the captured value from property development in R + P projects. Timing is also a critical factor for capturing the values. MTRC receives a ‘front end’ payment for land and a ‘back end’ share of revenues through purchasing the development rights from the HKSAR government at a ‘before rail’ price and sells these rights to the developers at an ‘after rail’ price. Thus, the captured value is efficiently used for recovering transport infrastructure investment as well as making considerable profits. Eventually, a virtuous cycle of viable operation of the railway is ensured in Hong Kong along with a TOD through employing an efficient institutional framework discussed overhead into the integrated Rail and property development curtailing the significant amount of transaction cost arising out of the process [31].

The central thesis of the R + P model lies in the fact that it embodies an institutional and regulatory framework comprising a supportive government land use and transport strategy for the high-quality urban built environment despite the simple use of captured land value to subsidize transit development [15]. But every model has its constraints and not even one is perfect. While theoretical explanation, as well as practical experience from some cases (i.e., Hong Kong), demonstrates the remarkable success story of the model, it also encountered some contemporary complications (i.e., privatization of urban space, social and economic exclusion) which necessities resolving toward moving forward taking it as a collective model for urban development. To safeguard broader urban sustainability, the institutional setup of the land development models (e.g., integrated land use-transport model) should carefully consider the complexity of multiple functions in urban society and the development of spatially integrated inclusive public space securing the wider public interest [4]. Some recently developed TOD practices enable planners and decision-makers to get around some institutional obstacles and accomplish a certain level of integrated development [42].

A wide variety of R + P projects already exist in Hong Kong with successful implications. In almost all the cases, most focus has been laid on housing development, although all have some degree of commercial development. This particular research has also been primarily centred on exploring the effective rule structure governing the land development game taking the best-case example of Hong Kong. Despite overseas, examples and practices have also been incorporated, pinpointed focus on the Hong Kong case itself may somehow be considered a limitation of the research which can be overcome with a more scrutinized systematic literature review.

## 7 Conclusion and way forward

Responding to the key research questions addressed, based on the Transaction cost framework, new institutional economics, and empirical examples this study concludes that the land development process involves formulating, interpreting, and enforcing the formal and informal rules or institutional arrangements in governing the interaction and action of various players in the very process and thus the null hypothesis has been rejected. However, before mimicking the efficiency rules in a local context, planning, managing and financing the land development process should be understood within an explicit governance structure and the socio-political context of a place. To progress forward toward integrated transport-land use development models, the importance of this synergy should be elevated and understood from a broader comprehensive framework.

The integrated planning approach in Hong Kong like the MTRC adopted R + P model relies deeply on a growing urban economy, a healthy real estate market and the availability of government land allocation. With the growing demand for precious urban land with strict regulatory measures (i.e., environmental control issues), it will be difficult in a dense urban built-up area in finding new land for future development and the opportunity to expand the R + P model. Moreover, despite providing a better institutional mechanism for governing effective property development process in countries like Hong Kong, escalating demand for subsidized and affordable housing units leads to criticism for the privatization of urban space as well as allocating development sites to the MTRC at prime locations for private property development [43]. Despite contributing to urban efficiency, privatization of urban space cannot be a total solution. Social inequality and exclusion, and fragmentation of urban space may occur due to the agglomeration of geographical advantages for transit-oriented development [[44], [45], [46]]. Potentially remarkable benefits can be added from encompassing transit‐value capture models, like R + P, to fast‐growing parts of the world, predominantly those in Asia [[14], [45], [47], [48]]. To sum up, when articulating, and enforcing the efficiency rules for governing key players' interactions and actions, all these contraventions should be given due attention for an optimum outcome arising out of the development process. As a prime limitation, the concerned researchers could incorporate more variables in the models used in this research to include diversified dimensions and a better understanding of the model. Future researchers are encouraged to work on this gap and contribute to this research argument.

## Data availability

Some or all data, models, or code generated or used during the study are available in a repository online in accordance with funder data retention policies. Some or all data, models, or codes that support the findings of this study are available from the corresponding author upon reasonable request. All data, models, and code generated or used during the study appear in the submitted article.

## Author contribution statement

Anutosh Das: Conceived and designed the experiments; Performed the experiments; Analyzed and interpreted the data; Contributed reagents, materials, analysis tools or data; Wrote the paper.

Sumita Roy: Contributed reagents, materials, analysis tools or data; Wrote the paper.
